# Efficient Sensing of Infected Cells in Absence of Virus Particles by Blasmacytoid Dendritic Cells Is Blocked by the Viral Ribonuclease E^rns^

**DOI:** 10.1371/journal.ppat.1003412

**Published:** 2013-06-13

**Authors:** Sylvie Python, Markus Gerber, Rolf Suter, Nicolas Ruggli, Artur Summerfield

**Affiliations:** Institute of Virology and Immunology, Mittelhäusern, Switzerland; University of California, San Diego, United States of America

## Abstract

Plasmacytoid dendritic cells (pDC) have been shown to efficiently sense HCV- or HIV-infected cells, using a virion-free pathway. Here, we demonstrate for classical swine fever virus, a member of the *Flaviviridae*, that this process is much more efficient in terms of interferon-alpha induction when compared to direct stimulation by virus particles. By employment of virus replicon particles or infectious RNA which can replicate but not form *de novo* virions, we exclude a transfer of virus from the donor cell to the pDC. pDC activation by infected cells was mediated by a contact-dependent RNA transfer to pDC, which was sensitive to a TLR7 inhibitor. This was inhibited by drugs affecting the cytoskeleton and membrane cholesterol. We further demonstrate that a unique viral protein with ribonuclease activity, the viral E^rns^ protein of pestiviruses, efficiently prevented this process. This required intact ribonuclease function in intracellular compartments. We propose that this pathway of activation could be of particular importance for viruses which tend to be mostly cell-associated, cause persistent infection, and are non-cytopathogenic.

## Author Summary

Plasmacytoid dendritic cells (pDC) represent the most potent producers of interferon type I and are therefore of major importance in antiviral defences. A TLR7-dependent induction of interferon-α in pDC by infected cells in the absence of virions has been demonstrated for hepatitis C virus. Here, we show that this pathway is also very efficient for classical swine fever virus, a pestivirus that is also a member of the *Flaviviridae*. Our data indicate a transfer of RNA from the donor cell to pDC in a cell-contact-dependent manner requiring intact lipid rafts and cytoskeleton of the donor cell. Importantly, we demonstrate that the enigmatic viral E^rns^ protein unique to pestiviruses efficiently prevents this pathway of pDC activation. This novel function of E^rns^ is dependent on its RNase activity within intracellular compartments. The present study underlines the importance of pDC activation by infected cells and identifies a novel pathway of virus escaping the interferon system. Considering that E^rns^ is required for pestiviruses to establish persistent infection of foetuses after transplacental virus transmission resulting in the development of immunotolerant animals, this report also points on a possible role of pDC in preventing immunotolerance after viral infection of foetuses.

## Introduction

Although representing a rare cell type of the immune system, plasmacytoid dendritic cells (pDC) are the most important source of systemic interferon (IFN) type I in the early phase of many virus infections, and as such a critical early alarm system against viruses [1], [2]. This is based on the ability to produce around 1000 times more IFN type I than any other cell type [1]. Accordingly, pDC possess the necessary cell biological features such as Toll-like receptor (TLR)7 and TLR9 and constitutive high levels of IFN regulator factor (IRF)-7 to sense viruses with high efficiency [2]. While it is evident from the literature that TLR7 is the most important sensor of RNA viruses and TLR9 for DNA viruses, it is less clear how the viral nucleic acids have access to these compartments and how the encapsulated viral nucleic acids get in contact with the TLR's. Recently, a novel process of pDC stimulation by infected cells independent of viral particles and their uptake by pDC has been described for hepatitis C virus (HCV) in which pDC sense infected cells in a cell contact- and TLR7-dependent manner [3], [4]. This process has been described to be more effective than stimulation of pDC with cell-free virions. Stimulation of pDC by infected cells has also been reported for HIV [5]. However, this process was blocked by neutralizing anti-envelope antibodies [5] implying a different mechanism of RNA transfer to the TLR7 compartment as observed with HCV where stimulation is not blocked by virus neutralization [3]. For the latter virus, lipid rafts and tetraspanin-enriched membrane microdomains have been described to be involved in infected cell sensing by pDC [4].

Considering the potential importance of this mechanism of pDC activation, we initiated this study with a focus on another member of the *Flaviviridae*, classical swine fever virus (CSFV) belonging to the pestivirus genus. CSFV is the causative agent of a viral hemorrhagic fever in pigs with disease characteristics resembling dengue hemorrhagic fever if pigs are infected with highly virulent CSFV strains. However, with low virulent strains chronic disease and persistent infections are observed [6]. CSFV, in contrast to HCV, has a particular tropism for cells of the macrophage and DC lineage and most efficiently infects pDC [7]–[9].

Our results demonstrate that the basic characteristics of pDC stimulation by infected cells resemble those of HCV. In addition, using such cultures the present study identified a striking function of the enigmatic viral E^rns^, an essential structural protein with ribonuclease (RNase) activity [10]. The pestivirus genus is composed of major veterinary pathogens, the most important being CSFV and bovine viral diarrhea virus (BVDV). Interestingly, E^rns^ is unique to pestiviruses. Despite its function as structural protein, E^rns^ exists as soluble form secreted from infected cells and has been proposed to be involved in immune evasion of pestiviruses (for review, see [11]). Removal of the RNase activity was demonstrated to result in virus attenuation [12], [13] and abrogation of the capacity of pestiviruses to establish immune-tolerance and persistent infections after infection of fetuses [14], [15]. However, it has been difficult to understand the mechanism of immune evasion using *in vitro* studies. Recombinant E^rns^ degrades synthetic single-stranded and double-stranded RNA added to the cultures [16]–[18] but pestiviruses with or without RNase activity do not induce IFN type I in cell culture and replicate to the same titers as their wild type counterpart. In this study we have identified how E^rns^ potently counteracts IFN-α induction in pDC. It represents the first example of a viral protein that prevents the stimulation of pDC by infected cells, and thus represents a novel pathway of viral evasion of the type I IFN system. Furthermore, it underlines the importance of stimulation of pDC by infected cells, rather than virions.

## Results

### Infected cells represent a powerful activator of IFN-α responses by pDC

**Figure 1:**
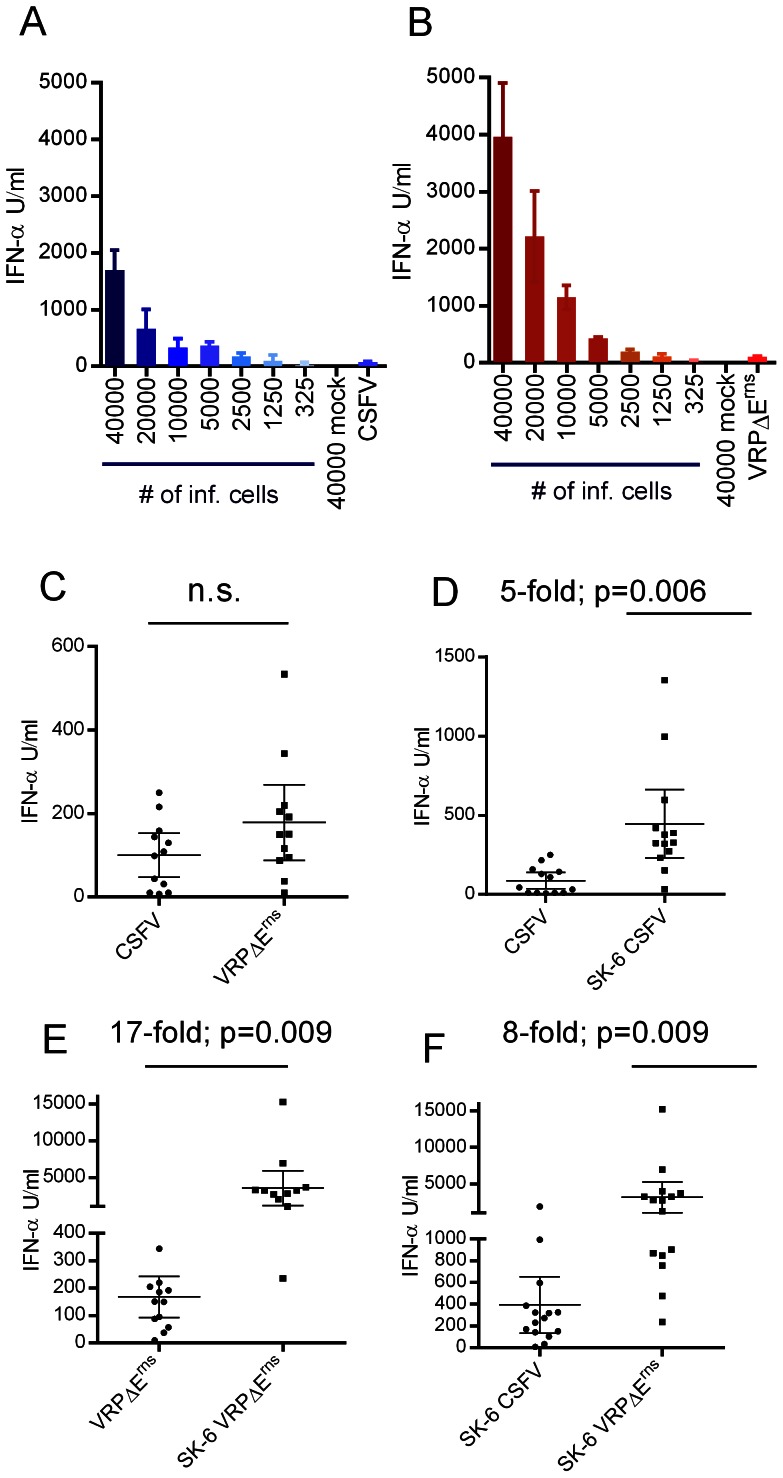
CSFV-infected cells are powerful inducers of INF-α response by pDC. Enriched pDC were either stimulated with CSFV-infected SK-6 cells, VRPΔE^rns^-infected SK-6 cells or directly by CSFV or VRPΔE^rns^ infection. IFN-α was quantified by ELISA after 22 h of culture. A and B. Cell number-dependent IFN-α induction in pDC by CSFV- or VRP-infected SK-6 cells, respectively. The number of infected cells (# of inf. cells) or mock treated cells added to 200'000 CD172a^+^ enriched pDC per microwell is indicated. The last bars, labeled CSFV (A) and VRPΔE^rns^ (B), show the IFN-α levels induced by direct virus stimulation. In C–F, the results of 12–15 independent experiments with standard deviations, fold increase calculated from the means of the compared groups and p values of paired students t-test are shown (n.s.: not significant). C. IFN-α induced by direct stimulation with either CSFV or VRPΔE^rns^. D. IFN-α induced by stimulation with either CSFV or CSFV-infected SK-6 cells. E. IFN-α induced by stimulation with either VRPΔE^rns^ or VRPΔE^rns^-infected SK-6 cells. F IFN-α induced by stimulation with cells infected with either CSFV or VRPΔE^rns^. The purity of pDC 2–5%.

In accordance to previous studies [7], [8], CSFV as well as virus replicon particles (VRP) lacking the E^rns^ gene (VRPΔE^rns^) were poor stimulators of pDC , inducing between 0 and 550 IFN-α units per ml, dependent on the experiment. Interestingly, stimulation of pDC by co-culture with CSFV-infected or VRPΔE^rns^ -infected SK-6 cells induced up to 100-fold more IFN-α compared with direct infection of the pDC, with an optimum at 40'000 to 80'000 infected SK-6 cells per 2×10^5^ CD172a^+^ enriched pDC (Figure 1A and B). While no significant difference between direct CSFV and VRPΔE^rns^ stimulation was observed, CSFV-infected SK-6 cells stimulated an average of 5.1 more IFN-α when compared to direct stimulation by CSFV (Figure 1C and D). This difference was even more evident when direct simulation with VRPΔE^rns^ was compared to stimulation by VRPΔE^rns^-infected cells (Figure 1E). Interestingly, VRPΔE^rns^-infected SK6 cells were in average around 8 times more stimulatory than CSFV-infected SK6 cells (Figure 1F).

In accordance to previous studies demonstrating that pDC were the only cell type able to respond to CSFV by IFN-α production [19]–[21], pDC were the only source of IFN-α following stimulation with infected cells, as demonstrated by intracellular IFN-α staining which was only found in the CD4^high^CD172a^+^ pDC population. Furthermore, purified monocytes did not produce IFN-α in response to any of the stimuli tested (Supplementary Figure 1).

### Infectious RNA or viral protein is transferred between infected cells and pDC/monocytes

**Figure 2:**
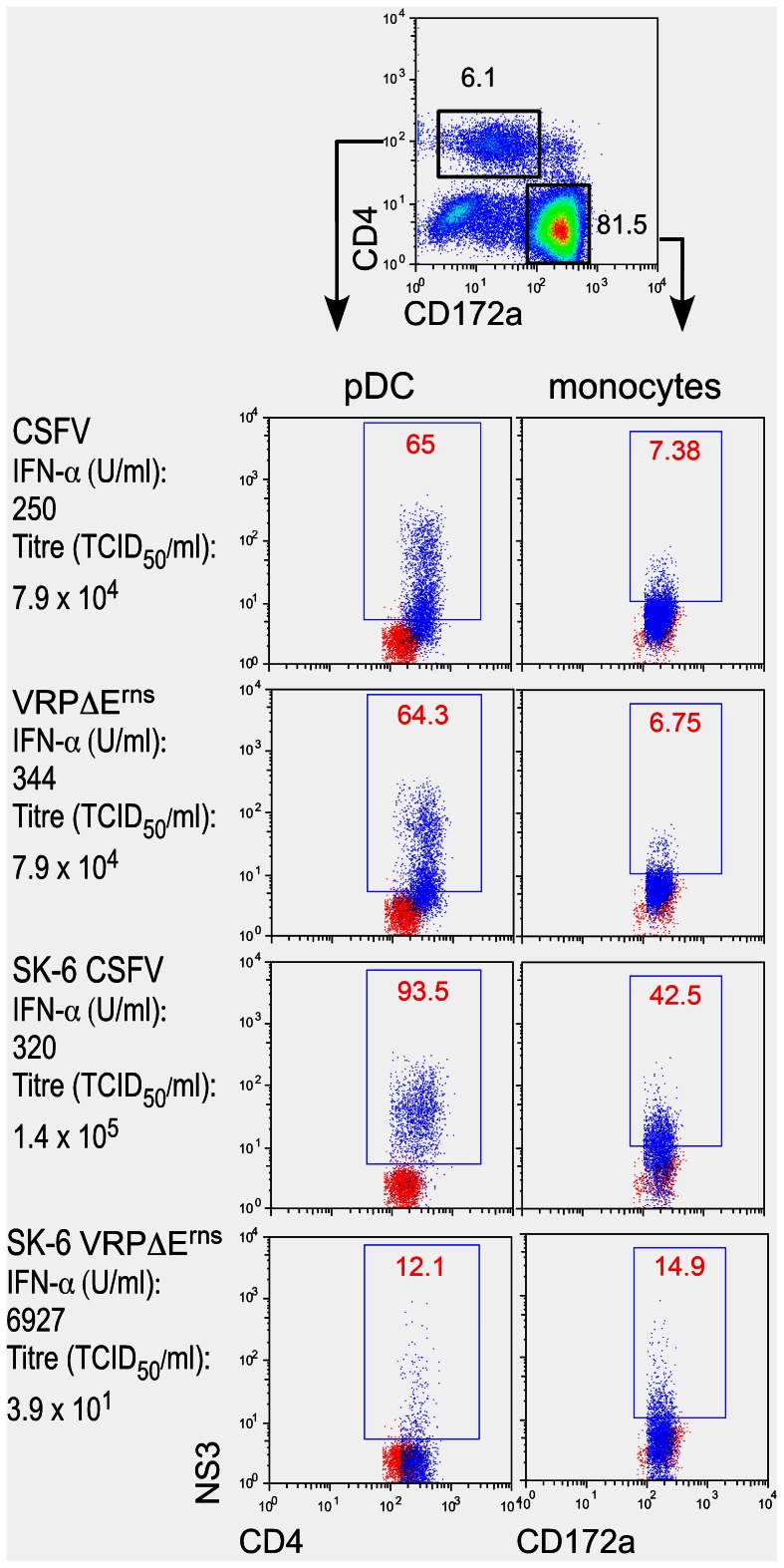
Viral protein expression in pDC and monocytes after co-culture with VRP-infected cells. Enriched pDC were either infected with CSFV, VRPΔE^rns^ or co-cultured with CSFV- or VRPΔE^rns^-infected SK-6 cells for 20 h, and then analyzed by three-color FCM to determine the NS3 expression in pDC (defined as CD4^+^CD172a^low^, left side dot plots) and monocytes (defined as CD4^−^CD172a^high^, right side dot plots). For the CD4/NS3 and CD172a/NS3 dot plots, the blue dots represent NS3 staining of infected cultures overlaid on red dots representing the NS3 staining on non-infected cultures. The percentage of NS3 cells is shown in red for the infected cultures. On the left side the mean IFN-α values and viral titers measured in the cultures are shown. The results are representative of triplicate cultures repeated in three independent experiments.

The above results suggested that infected SK-6 cells would transfer viral RNA to pDC resulting in pDC activation. Considering the fact that VRP deliver self-replicating RNA which replicates for many days in SK-6 cells [22], we tested if functional replicon RNA was transferred between SK-6 cells and pDC by determining the expression of the viral NS3 protein in pDC. NS3 is generated by post-translational processing of the CSFV precursor polyprotein. Detectable amounts of NS3 in cells can only be obtained with replication competent pestivirus genomes, i.e. full-length genomes and replicons. As shown in Figure 2, after co-culture of pDC with VRPΔE^rns^-infected SK-6 cells for 22 h, approximately 12–14% pDC expressed NS3, indicating either a transfer of intact full-length replicon RNA or viral NS3 protein between the cells. Interestingly, co-culture of CSFV-infected cells with pDC resulted in a higher degree of infection (94%) compared to direct infection by the virus (65%). Another observation was that, when infectious CSFV or VRP were present, the percentage of NS3-expressing pDC was higher when compared to the monocytes that were co-purified using the CD172a selection. It is also noteworthy that NS3^+^ monocytes were found after co-culture with VRP-infected SK-6 cells. The results presented in Figure 2 also highlight that there is no correlation between infectious titers, percentage of viral protein expressing pDC and IFN-α responses. An over 20 times higher IFN-α response was found when pDC were stimulated with VRP-infected SK-6 cells in which infectious virus was barely detectable. We confirmed these results by employing RT-PCR to quantify the number of viral genome copies in these cultures. pDC were stimulated either directly with VRPΔE^rns^ or with VRPΔE^rns^-infected SK-6 cells, and then re-sorted from these cultures by CD172a MACS sorting. This demonstrated that around 0.1% of the total viral genome present in infected SK-6 cells was transferred to pDC/monocytes. Direct infection of enriched pDC was ∼6 times more efficient in delivering RNA but induced much lower IFN-α levels (Table 1).

**Table 1:** Relationship of IFN-α responses and viral RNA.

Cells	IFN-α U/ml)	Viral genome copies per 10^6^ cells	% RNA relative to SK-6 VRPΔE^rns^
SK-6 VRPΔE^rns ^ a	0	44.8±8.4×10^6^	100
pDC MOCK^b^	0	0	-
pDC VRPΔE^rns^ b	18±1	2.8±0.3×10^5^	0.625
pDC SK-6 VRPΔE^rns^ c	4580±193	4.7±0.6×10^4^	0.105

a RNA was extracted from VRPΔE^rns^-infected (MOI 5 TCID_50_/cell, 24 h) SK-6 cells before co-culture.

b RNA was extracted from re-sorted enriched pDC (>99% CD172a^+^) treated with mock or infected withVRPΔE^rns^ (MOI 5 TCID_50_/cell, 22 h).

c RNA was extracted from re-sorted enriched pDC co-cultured with VRPΔE^rns^-infected SK-6 cells.

SK-6 cells or enriched pDC were infected with VRPΔE^rns^ at an MOI 5 TCID_50_/cell and IFN-α in supernatants and viral RNA quantified after 24 h. In the last row, SK-6 cells infected with VRPΔE^rns^-infected for 24 h were co-cultured with enriched pDC for further 22 h before IFN-α and viral RNA quantification. The latter used CD172a-re-sorted enriched pDC to remove contaminating SK-6 cells.

### Infected cell stimulation by pDC is not mediated by cell-free virions

**Figure 3:**
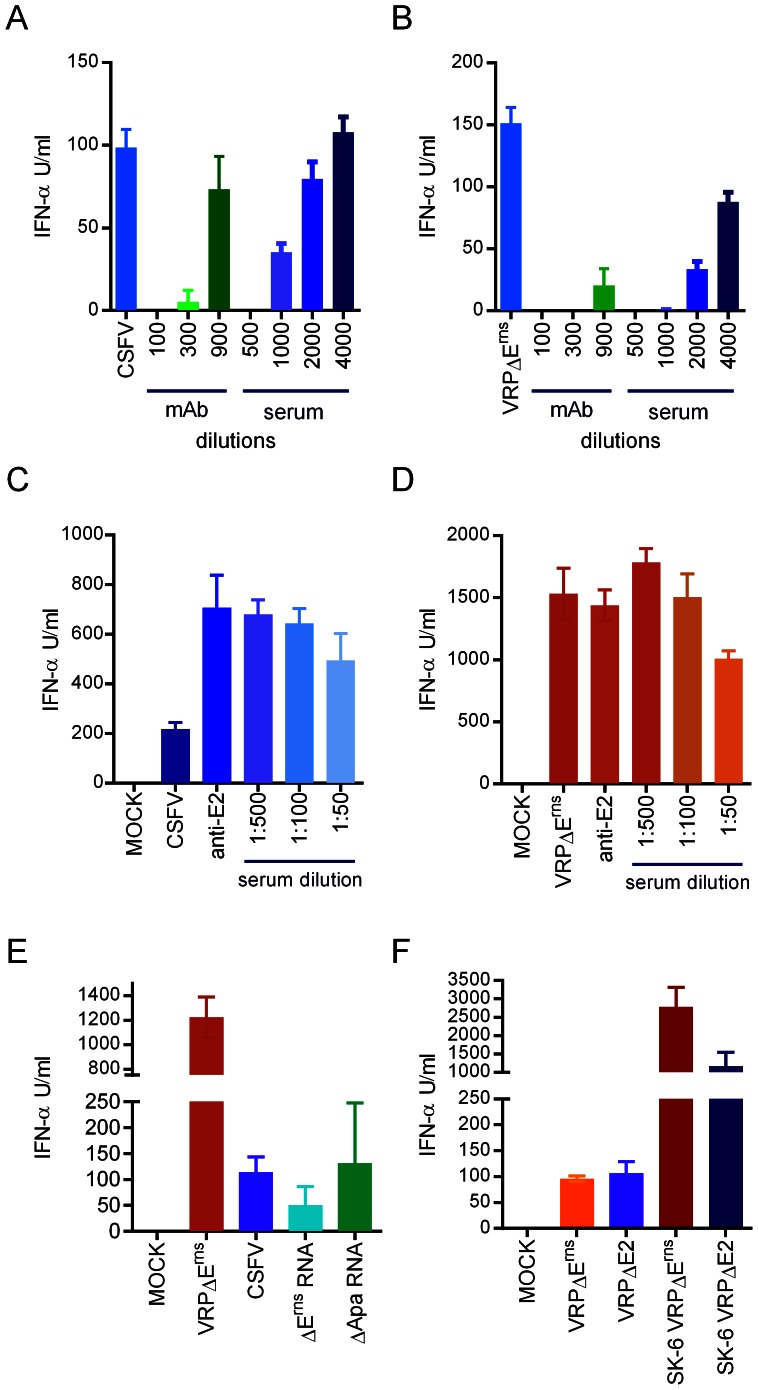
Stimulation of pDC by infected cells is not mediated by virions. A and B. Blocking of direct CSFV (A) or VRPΔE^rns^ (B) activation of pDC by addition of neutralizing antibodies. Results for different dilutions of anti-E2 glycoprotein mAb and of anti-CSFV serum are shown. C and D. The same antibodies (anti-E2 at 1∶40 dilution) did not reduce activation of pDC by CSFV- (C) or VRPΔE^rns^-infected (D) cells. The results are representative of four (A, B) or three (C–F) independent experiments. E. Cells transfected with self-replicating RNA unable to form virions stimulate pDC. *In vitro* transcribed viral RNA lacking the E^rns^ gene (ΔE^rns^ RNA) or all genes encoding the structural proteins of CSFV (ΔApa RNA) were co-cultured with enriched pDC for 20 h and IFN-α was determined. VRP- and CSFV-infected SK-6 cells were used as control (labeled VRP and CSFV). F. Stimulation of enriched pDC with either VRPΔE^rns^, VRPΔE2, SK-6 cells infected with VRPΔE^rns^ or SK-6 cells infected with VRPΔE2. For all experiments the mean of triplicate cultures with standard deviations are shown.

In order to rule out a role for free virions in the induction of IFN-α, we measured the infectivity in VRPΔE^rns^-infected SK-6 at the time of co-culture with pDC and found titers below 10^2^ TCID_50_/ml. This corresponded to MOI of less than 10^−4^ TCID_50_/enriched pDC, suggesting that stimulation of pDC does not occur by infection of pDC with VRP. The lack of involvement of free virions in pDC stimulation by infected cells was further confirmed by addition of a neutralizing monoclonal antibody against the main glycoprotein E2 of CSFV or by addition of a porcine polyclonal hyper immune serum obtained from an infected pig which contained antibodies against both glycoproteins of the virus, E2 and E^rns^. The antibody preparations completely blocked direct activation of pDC by cell-free virions (Figure 3A and B), but were unable to inhibit activation by infected cells, independent whether the stimulation used CSFV- or VRPΔE^rns^-infected SK-6 cells (Figure 3C and D). With CSFV-infected SK-6 cells, the antibodies even enhanced pDC stimulation. Antibodies from naïve animals or an irrelevant monoclonal antibody had no effect (data not shown). Similarly, the neutralizing antibodies blocked infection of pDC after direct stimulation but were unable to inhibit the expression of NS3 in pDC after stimulation by infected cells (supplementary Figure S2).

We further confirmed the absence of any virus particle in pDC stimulation by employing RNA-transfected SK-6 cells to stimulate pDC. To this end, SK-6 cells were transfected with *in vitro* transcribed RNA synthesized with plasmids encoding the E^rns^-deleted genome of CSFV (pA187-ΔE^rns^, ΔE^rns^ RNA) or a genome of CSFV devoid of all structural proteins (pA187Δ-Apa, ΔApa RNA). The results shown in Figure 3E demonstrate that CSFV RNA-transfected SK-6 cells can activate pDC, although the levels of IFN-α were low, compared to VRP-infected SK-6 cells. This was explainable by the relatively low transfection efficiency of 15–20% in terms of NS3^+^ expression (data not shown). We also generated VRP with a deletion of E2 instead of E^rns^. SK-6 cells infected with such VRPΔE2 were more potent in inducing IFN-α in pDC than VRPΔE2 virions. Interestingly, SK-6 cells infected with VRPΔE^rns^ were more stimulatory than SK-6 cells infected with VRPΔE2 (Figure 3F).

### Sensing of infected cells is reduced by a TLR7 inhibitor

**Figure 4:**
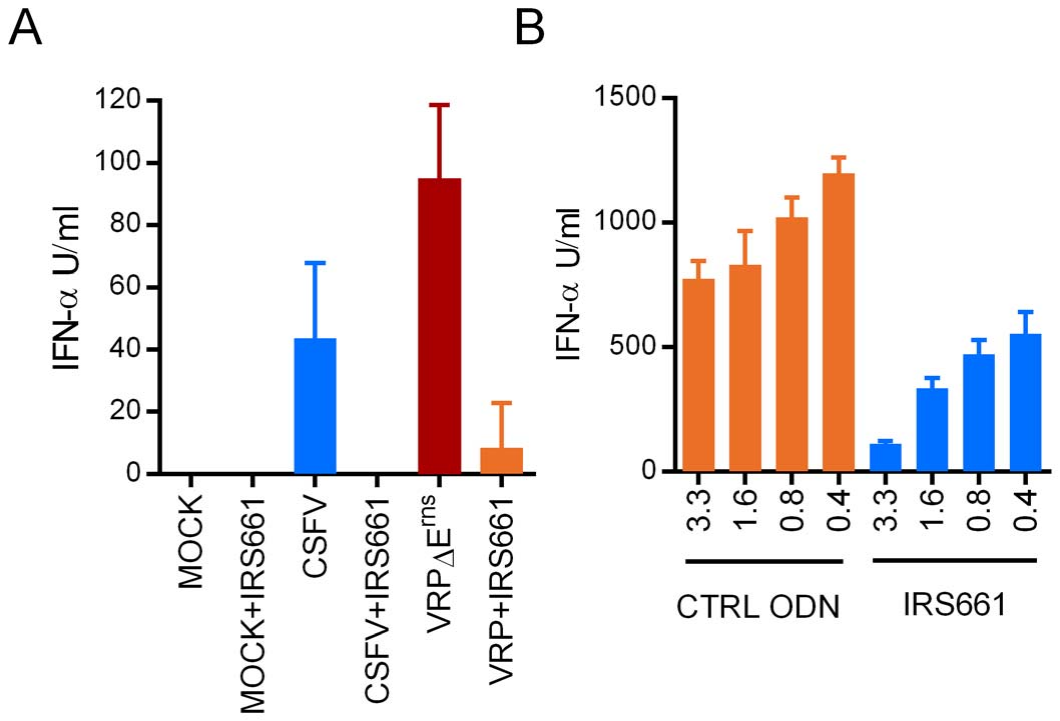
Stimulation of pDC by infected cells is mediated by TLR7. IRS661 inhibits pDC activation by direct CSFV or VRPΔE^rns^ infection (A) and by VRP-infected cells (B). In A, IRS661 was employed at 1.7 µM, and in B, at the indicated µM concentrations. In B, the IFN-α levels of the cultures treated with IRS661 were significantly lower (p<0.05) than those receiving control (CTRL) ODN at all concentrations. The bars represent the mean values of three replicates, with error bars showing the standard deviations. The results are representative of three independent experiments.

In order to determine the role played by TLR7 in sensing CSFV-infected cells, we used the immunoregulatory sequence 661 (IRS661) representing an oligodeoxynucleotide inhibitor of TLR7 which had been previously established for the human, murine and porcine immune systems [23], [24]. IRS661 at a concentration of 0.7 µM efficiently inhibited CSFV- and VRP-induced pDC activation (Figure 4A). With infected cells inducing much higher levels of IFN-α, the reduction caused by IRS661 was still over 80% with the highest inhibitor concentration (Figure 4B).

### The viral ribonuclease E^rns^ efficiently inhibits pDC stimulation by infected cells

**Figure 5:**
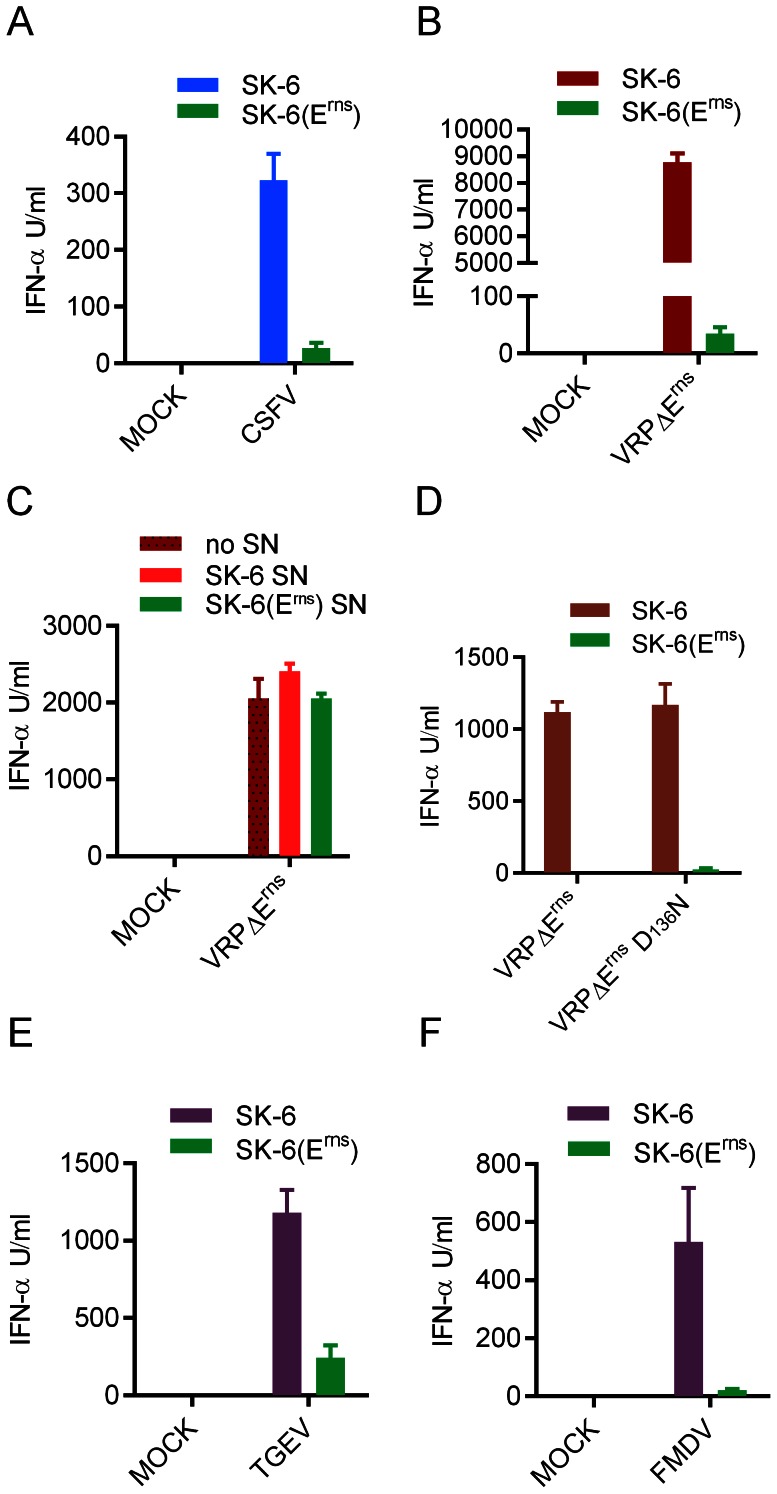
E^rns^ disables stimulation of pDC by infected cells. A and B. IFN-α induction in pDC after stimulation with SK-6 or SK-6(E^rns^) cells infected with CSFV (A) or VRPΔE^rns^ (B). C. Lack of inhibitory activity in supernatants (SN) of SK-6(E^rns^) cells. Supernatants harvested from SK-6 or SK-6(E^rns^) cell cultures were added to co-cultures of enriched pDCs with MOCK- or VRPΔE^rns^-infected SK-6 cells at 50% (V/V) D. N^pro^ has no impact on stimulation of pDC by infected cells. SK-6 or SK-6(E^rns^) cells were infected with wild type VRPΔE^rns^ or VRPΔE^rns^ with the D_136_N mutation in N^pro^ (VRPΔE^rns^ D_136_N) abrogating the functional activity of N^pro^ in preventing IRF3 and IRF7-mediated IFN type I responses. E and F. E^rns^ also inhibits the activation of pDC by SK-6 cells infected with TGEV (E) and FMDV (F). SK-6 or SK-6(E^rns^) cells were infected with TGEV (MOI of 10 TCID_50_/cell), or FMDV (MOI of 2.5 TCID_50_/cell), cultured for 90 min followed by addition of enriched pDC. For A to F, IFN-α in the supernatants was quantified by ELISA after 20 h of co-culture. The mean values of three replicates with standard deviation are shown. In A and B, the results are representative of five independent experiments. In C to F, the data is representative of two independent experiments.

**Figure 6:**
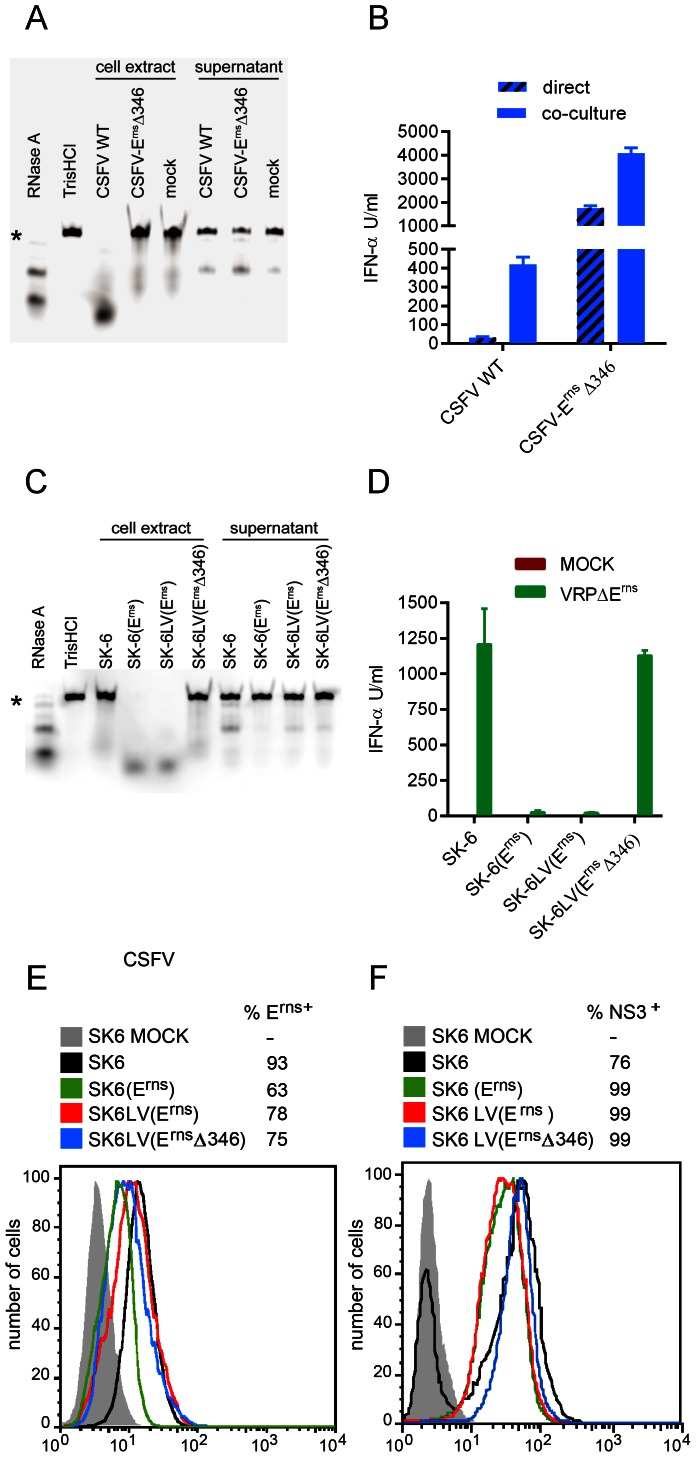
The RNase activity of E^rns^ is required for its inhibitory function. A and C. Deletion of histidine 346 abolishes the RNase activity of the viral E^rns^ in virus-infected cells (A) and in stable cell lines expressing the glycoprotein (C). Confluent monolayers of SK-6 cells infected for 24 h with CSFV vA187-1 (CSFV WT) or with the mutant vA187-E^rns^Δ346 virus (CSFV-E^rns^Δ346) (A), and of the cell lines SK-6, SK6(E^rns^), SK-6LV(E^rns^) and SK-6LV(E^rns^Δ346) (C) were washed 5 times with phosphate buffered saline and cultured for another 16 h in serum-free MEM. The culture supernatants were then collected and the cell extracts obtained by hypotonic lysis with H_2_O. The Dy-781-O1-RNA probe was incubated with MEM containing RNase A, with TrisHCl, and with cell extract or supernatant of cells infected with CSFV WT, CSFV-E^rns^Δ346 or mock (A) or of SK-6, SK6(E^rns^), SK-6LV(E^rns^) and SK-6LV(E^rns^Δ346) cells (C), and separated by urea/polyacrylamide gel electrophoresis. The star indicates the position of undigested RNA probe. B. CSFV with inactive RNase function of E^rns^ (CSFV-E^rns^Δ346) is a better stimulator of pDC. Enriched pDC were activated by virus (hatched bars) or by co-culture with infected SK-6 cells. The IFN-α responses to wild type CSFV and CSFV-E^rns^Δ346 are compared. D to F. SK-6 cells expressing RNase active E^rns^ prevent IFN-α induction by VRPΔE^rns^ infection, while VRPΔE^rns^-infected SK-6 cells expressing E^rns^Δ346 re-gain their ability to stimulate pDC. SK-6 cells were transduced by lentiviral gene delivery to express wild type E^rns^ or E^rns^Δ346 with 75% transduction efficiency (E). The SK-6(E^rns^), and the LV-transduced SK-6LV(E^rns^) and SK-6LV(E^rns^Δ346) were infected with VRPΔE^rns^ and then tested for their ability to induced IFN-α in enriched pDC (D). The mean values of three replicates with standard deviations are shown calculated from data which is representative of three independent experiments. All E^rns^ expressing cells lines were highly susceptible to infection by VRPΔE^rns^ as determined by viral NS3 expression (F).

A striking observation was that in all experiments VRPΔE^rns^-infected SK-6 cells were clearly more efficient at inducing IFN-α compared to CSFV-infected SK-6 cells (Figure 1F). Considering that VRPΔE^rns^ lacks the E^rns^ gene, we postulated a role for this viral protein in inhibition of this novel type of IFN-α induction and tested this by comparing SK-6 cells stably expressing E^rns^ to the parent wild type SK-6 cells. Indeed, the SK-6(E^rns^) cells infected with VRP or CSFV were very inefficient at inducing IFN-α in contact with pDC (Figure 5A and B). This was not a result of different susceptibility to the viruses as the infection rate of the SK-6(E^rns^) was 99%, similar to CSFV (Figure 6F). As expected, E^rns^ expressed by the SK-6(E^rns^) trans-complemented VRPΔE^rns^ to generate infectious particles resulting in a higher degree of NS3^+^ pDC (supplementary Figure S3). Considering that this must be associated with a higher viral RNA load in pDC, these results indicate a potent function of E^rns^ in preventing the stimulation of pDC by infected cells.

E^rns^ has been shown to be mainly associated with intracellular membranes in particular of the ER, with almost no cell surface expression [25]. However, considering that approximately 16% is secreted to the extracellular space [26], [27], we tested if E^rns^ secreted by SK-6(E^rns^) cells was responsible for the observed inhibition. To this end, supernatants of SK-6(E^rns^) or SK-6 were added to co-cultures of VRPΔE^rns^-infected SK-6 cells and pDC. As shown in Figure 5C, there was no evidence for any suppressive effect of soluble E^rns^ in the supernatants.

We next determined the role of N^pro^, a well established type I IFN antagonist of CSFV in non-pDC and pDC targeting IRF3 and IRF7 transcription factors [8], [19], [28]. To this end, we compared the ability of SK-6 cells infected with VRPΔE^rns^ and VRPΔE^rns^ D_136_N expressing a non-functional mutant of N^pro^ [22], to activate pDC. Our results demonstrated that only E^rns^, but not N^pro^ prevents the activation of pDC by infected cells (Figure 5D).

In order to confirm this function of E^rns^, we compared the effect of SK-6 and SK-6(E^rns^) cells infected with two other RNA viruses, the picornavirus foot-and-mouth disease virus (FMDV) and the coronavirus transmissible gastroenteritis virus (TGEV), on pDC. When the stimulation used FMDV- or TGEV-infected SK-6 and SK-6(E^rns^) cells for comparison, the responses with infected SK-6(E^rns^) cells were 3 to 6-fold lower compared to SK-6 cells (Figure 5 E and F). This was not caused by an inhibitory effect of E^rns^ on virus replication (Supplementary Figure S4). In order to exclude a potential “toxicity” derived from E^rns^ expressing cells we also tested the responses of pDC to CpG when stimulated in co-cultures with SK-6 and SK-6(E^rns^) cells, and found similar levels of IFN-α (Supplementary Figure S5).

### The ribonuclease activity of E^rns^ is required to suppress infected cell-induced IFN-α

In order to test the requirement of RNase activity for the above E^rns^ function, we constructed an RNase-negative mutant of CSFV by deleting the histidin codon at position 346 [13]. The mutation abolished the RNase activity (Figure 6A), confirming previously published data [13]. Interestingly, RNase activity of E^rns^ was detectable in cell extracts only (Figure 6A). While SK-6 cells infected with wild type CSFV induced under 500 U/ml of IFN-α, cells infected with the CSFV-E^rns^Δ346 mutant induced approximately 10-fold higher responses (Figure 6B). Furthermore, direct stimulation of pDC by virus also dramatically increased when the CSFV-E^rns^Δ346 mutant was employed. Nevertheless, the levels of IFN-α remained clearly under those induced by infected cells. For further confirmation that the inhibitory effect of E^rns^ in pDC depends on the RNase-activity of E^rns^, we constructed lentivirus-transduced SK-6 cell lines expressing the parent E^rns^ [SK-6LV(E^rns^)] or an RNase-inactive mutant E^rns^ [SK-6LV(E^rns^Δ346)]. As expected, the deletion of the histidine at position 346 completely abolished the RNase activity of E^rns^ (Figure 6C). Again, RNase activity of the parent E^rns^ was detectable only in the cell extracts. As expected, only SK-6 expressing RNase active E^rns^ prevented IFN-α induction by VRP infection of the cell lines (Figure 6D). All SK-6 cell lines expressing both wild-type and mutant E^rns^ had comparable levels of E^rns^, demonstrating that the striking differences observed in RNase and inhibitory activity for activation of pDC were not caused by lack of E^rns^ expression. In addition, the levels of E^rns^ in these cells were below those found after natural infection by wild-type CSFV (Figure 6E). Furthermore, all E^rns^-expressing cell lines were found to be fully susceptible to VRPΔE^rns^ infection as determined by NS3 expression (Figure 6F). The observed higher levels of NS3 expression in E^rns^ expressing cells was a consequence of trans-complementation of VRPΔE^rns^ and generation of infectious virions in these cultures.

**Figure 7:**
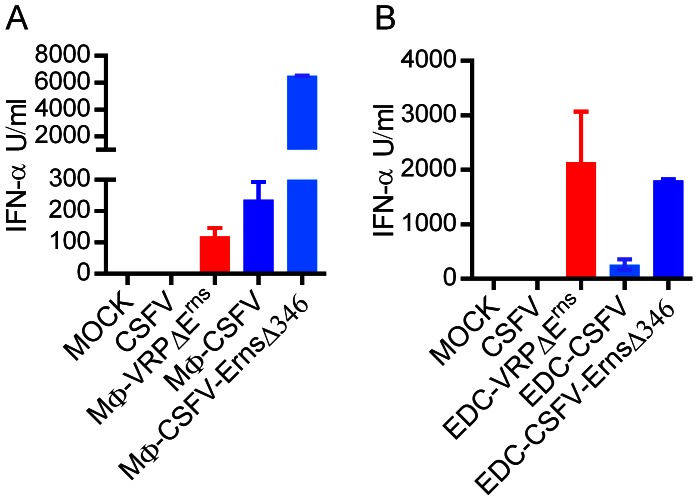
Stimulation of pDC by infected MΦ and EDC. MΦ (A) or PEDSV.15 cells representing an immortalized EDC line (B) were infected with either VRPΔE^rns^, CSFV, or CSFV-E^rns^Δ346 (MOI 5 TCID_50_/cell) and incubated for 48 h before co-culture with enriched pDC. The responses are compared to direct stimulation by CSFV or VRPΔE^rns^. Mean and standard deviation calculated from triplicate cultures are shown. The results are representative of two independent experiments.

Considering that macrophages (MΦ) and endothelial cells (EDC) represent important target cells for CSFV, we also tested if infected MΦ and EDC, similar to SK-6 cells, were able to activate pDC. Indeed, VRPΔE^rns^-infected and CSFV-infected MΦ were able to activate pDC while no responses were detectable with CSFV alone (Figure 7A). VRPΔE^rns^ were relatively inefficient at infecting MΦ (26% NS3^+^ versus 97% NS3^+^ with CSFV), which explains the lower IFN-α responses when compared to CSFV. The highest levels of IFN-α were induced by MΦ infected with the CSFV-E^rns^Δ346 mutant (83% NS3^+^ MΦ) confirming the functioning of E^rns^ also in MΦ (Figure 7A). Neither the VRPΔE^rns^, nor WT CSFV or the CSFV-E^rns^Δ346 mutant were able to induce IFN-α in MΦ (data not shown). We also further confirmed our findings using the immortalized porcine EDC line PEDSV.15. VRPΔE^rns^-infected and CSFV-E^rns^Δ346-infected EDC were more potent at activating pDC when compared to CSFV-infected EDC (Figure 7B). In contrast to MΦ, the rate of endothelial cells infection was comparable with the viruses and VRPΔE^rns^ (VRPΔE^rns^: 80% NS3^+^, CSFV: 92% NS3^+^ and CSFV-E^rns^Δ346 mutant 92% NS3^+^).

### Stimulation of pDC by infected cells requires cell contact

**Figure 8:**
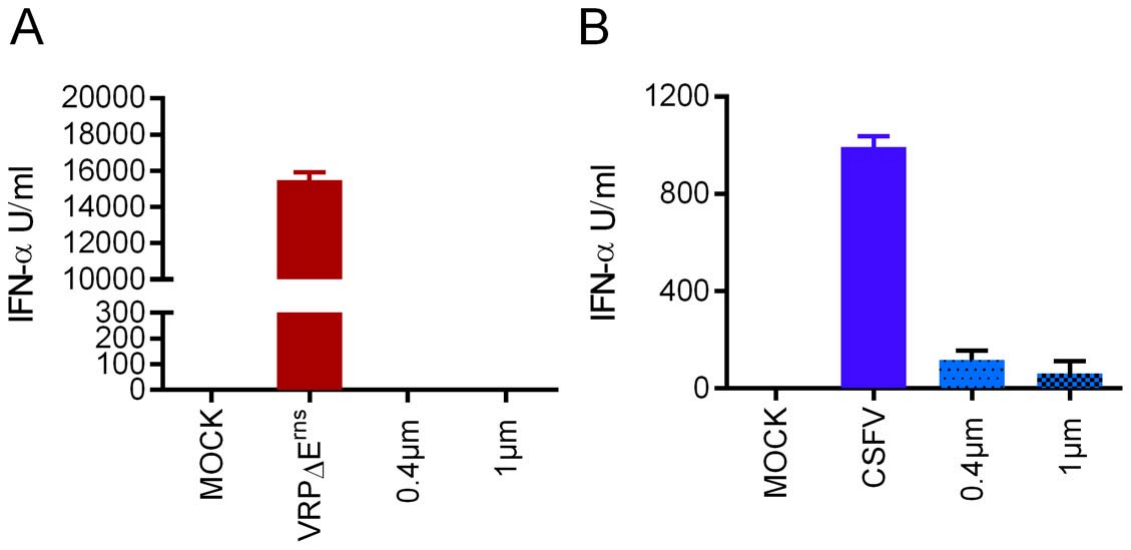
pDC stimulation by infected cells is cell contact-dependent. A and B. VRPΔE^rns^- (A) or CSFV- (B) infected SK-6 cells were co-cultured with enriched pDC for 24 h either in contact or separation using transwell inserts of 0.4 or 1 µm. The mean IFN-α values of three replicates measured by ELISA are shown with the standard deviation, calculated from a set of data which is representative of three independent experiments.

Based on the above results we postulated that viral RNA is transported from the infected cells to the TLR7 compartment of pDC in a manner avoiding contact of the RNA with the extracellular space. Consequently, we were interested to investigate if membrane vesicles transporting viral RNA from the infected cell to the pDC could be involved in pDC activation by infected cells. To this end, we co-cultured pDC with VRP-infected SK-6 cells in transwell culture dishes using 0.4 or 1 µm pore sizes. Only pDC with direct contact to infected SK-6 cells produced large quantities of IFN-α (Figure 8A and B). Notably, pDC responses to the CpG control were in the same level of magnitude if the pDC were cultured in the insert or in the well of the plate (data not shown). When the same experiments were performed with CSFV-infected SK-6 cells, low IFN-α responses were also observed in all transwell conditions (Figure 8B). Considering that CSFV virions are only 60 nm, this response was probably mediated by direct stimulation of pDC with virions passing the membranes. Similar to VRP-infected or CSFV-infected SK-6 cells, infected MΦ and EDC were unable to induce IFN-α when separated from pDC using a transwell culture system (data not shown).

#### Drugs affecting cytoskeleton and membrane cholesterol prevent stimulation of pDC by infected cells

**Figure 9:**
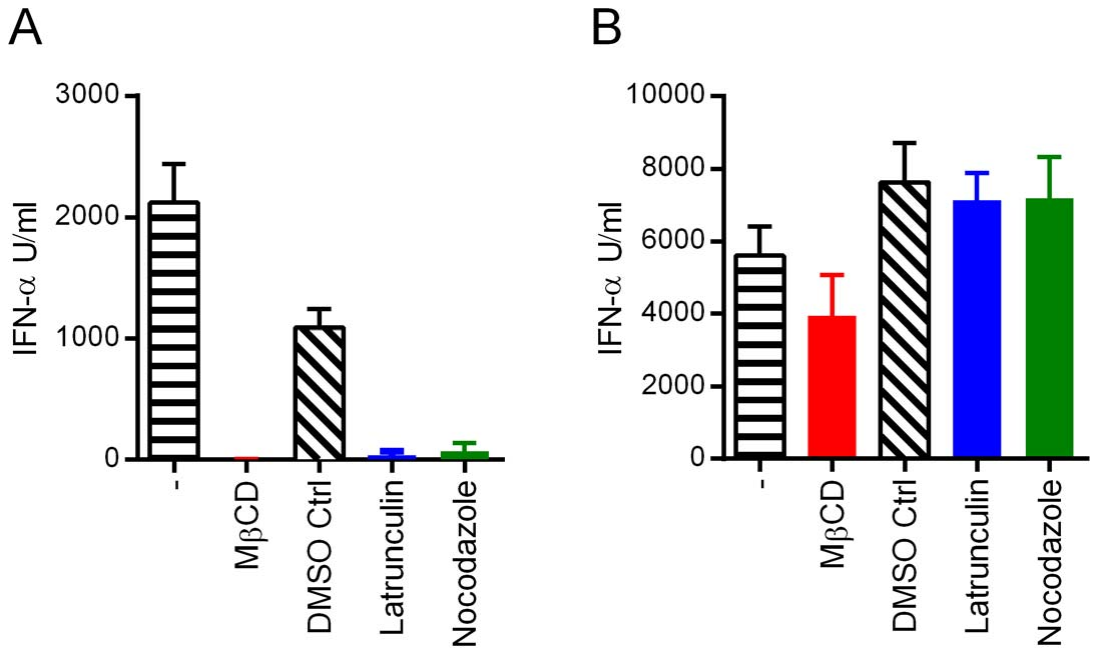
Drugs affecting cytoskeleton and membrane cholesterol prevent stimulation of pDC by infected cells. A. SK-6 cells were infected with VRPΔE^rns^ for 24 h, washed and then treated with a DMSO control, nocodazole (5 µM), MβCD (20 mM) or latrunculin (1 µM) for 2 h at 37°C. The inhibitors were then removed and the cells washed three times to avoid effects on pDC. The treated cells were co-cultured with pDC and IFN-α measured after 20 h of incubation. B. Uninfected SK-6 cells were treated as in A and co-cultured with pDC in presence of CpG. The mean values of three replicates with standard deviation are shown. The data is representative of three independent experiments.

The above results indicate that infected SK-6 cells transfer RNA to pDC using a pathway which requires cell-to-cell contact dependent communication between infected cells and pDC. In order to further characterize this pathway we tested the impact of cytoskeleton inhibitors as well as the cholesterol-depleting chemical MβCD. The VPR-infected SK-6 cells were treated for the last 2 h of infection followed by removal of the inhibitor by a wash step before co-culture, to avoid direct effects of the drugs on pDC and to enable efficient virus replication before co-culture. As shown in Figure 9, latranculin B, an inhibitor of microfilament formation [29], as well as nocodazole, which depolymerizes microtubules [30], almost completely prevented pDC activation. Furthermore, depletion of cholesterol from SK-6 cells by MβCD treatment prior to co-culture with pDC also blocked IFN-α responses of pDC, suggesting a potential role of lipid rafts in RNA transfer to pDC. To rule out a direct suppressive effect of the drugs on pDC functions we also stimulated pDC with CpG in presence of drug-treated uninfected SK-6 cells, and found no significant effects (Figure 9B). Furthermore, we demonstrated that the drug treatments did no negatively impact virus replication in SK-6 cells (Supplementary Figure S6).

## Discussion

During a virus infection, pDC will not only encounter virions but also virus-infected cells. The ability to sense the latter has the advantage of being able to sense infection before or without release of virions and also to better sense viruses which are particularly cell-associated and tend to cause persistent virus infections, such as HIV, HCV and pestiviruses. The present study underlines the importance of this by demonstrating that pDC stimulation by infected cells can be much more efficient than stimulation by virions. This is also emphasized by the identification of a viral protein that appears to have evolved to efficiently inhibit this pathway. After HIV [5], HCV and Venezuelan equine encephalitis virus [3], CSFV is now the fourth virus for which this mechanism of pDC stimulation has been found to be more potent than direct stimulation of pDC by virions. Although free CSFV was also able to stimulate pDC, the IFN-α responses were inferior to those induced by cell-free virions. Interestingly, also with FMDV, a virus which does not or very inefficiently stimulate pDC [31], infected cells were able to stimulate pDC.

The basic characteristics of pDC stimulation by CSFV-infected cells are similar to those observed with HCV. It represents a TLR7-dependent process which cannot be blocked by neutralizing antibodies and does not require expression of viral glycoproteins [3]. This is in contrast to the situation for HIV [5]. Our data indicate that this pathway of pDC activation is dependent on cell contact, intact actin filaments, microtubules as well as cell membrane cholesterol indicating a role for lipid rafts, although future studies are required to directly demonstrate the functional interaction of these cellular components with viral proteins. We have also identified that the pestiviral E^rns^ potently prevents pDC activation by infected cells. E^rns^ possesses several remarkable features, of which the RNase activity is of particular interest, considering that it is expressed by an RNA virus. E^rns^ has structural similarities with plant T2 RNases which have their optimal catalytic activity at an acidic pH [32] with a preference for cleaving single-stranded RNA [33], [34]. This would point on an activity within the endosomal compartment, which is also supported by our data indicating that genomic RNA does not appear to be degraded. The protein also has an unusual membrane anchor composed of an amphipathic helix without a typical membrane anchor [27], [35], but with a retention signal ensuring its association with intracellular membrane compartments [25]. Whether this enables accumulation in the endosomal system with appropriate orientation needs to be investigated. We postulate that viral RNA may have access to the endosomal system via the autophagy process. These endosomes could be transferred to pDC by a pathway to be defined and then fuse with the TLR7 compartment. Alternatively, both cytoplasmic viral RNA and E^rns^ could be autophagocytosed after transfer to pDC followed by fusion of autophagosomes with TLR7-containing endosomes [36]. Only at this acidic location the RNAse function would be highly active and rapidly degrade the viral RNA to reduce TLR7 triggering. Consequently, one of the first questions to be addressed in future studies is whether E^rns^ is transported from SK-6 cells to pDC to degrade RNA in the TLR7 compartment of pDC. Certainly, E^rns^ can act in pDC as our data is showing that CSFV with E^rns^ lacking RNase activity induce much higher IFN-α responses compared to wild type CSFV when pDC are directly stimulated by virions. Strikingly, E^rns^ can exert its inhibitory function when expressed independently of the viral context in the infected cells that stimulate the pDC. In addition, our results suggest that E^rns^ functions by using intracellular rather than extracellular pathways, since SK-6(E^rns^) supernatants did not suppress pDC activation by VRPΔE^rns^-infected SK-6 cells. This is contrary to a role proposed for secreted E^rns^ by several authors. Based on the observation that a minor part of the protein was found to be secreted from infected cells or cells expressing E^rns^ [25]–[27], recombinant E^rns^ was tested and found to degrade synthetic single-stranded and double-stranded RNA added to the cultures [16]–[18].

Considering that pDC are by far the most potent producers of IFN type I and of crucial importance in linking innate to adaptive immunity, our data shed light into one of the most fascinating aspects of pestivirus biology. These viruses cause persistent infections, in both cattle and pigs. When bovine fetuses are infected transplacentally by BVDV between the 2^nd^ and 4^th^ month of pregnancy, a time point at which the fetuses are not yet immunocompetent [37], persistently infected calves are born which are fully immunotolerant to the virus and cannot mount any adaptive immune responses against the virus. These persistently infected calves play a major role in the epidemiology of the disease by shedding virus. Similar observations have been reported for pigs after infection of pregnant sows with low virulent strains of CSFV before eradication of CSFV from most European countries and the U.S.A. [38]–[44]. This contrasts with the pathogenesis of highly virulent strains of CSFV which induce acute disease with high mortality which is associated with high systemic levels of IFN-α [45]. This appears contradictory to the present report but compared to other viruses the ability of CSFV to activate pDC is weak [9]. Even if pDC are stimulated by cells infected with wild type CSFV, IFN type I responses remain relatively weak compared to influenza virus. Our concept to explain this apparent contradiction to the *in vivo* situation with virulent strains of CSFV is based on the prominent tropism of CSFV for MΦ and DC, and its localization predominantly in lymphoid tissue. Highly virulent isolates of CSFV cause a very rapid and strong viremia, in which the virus reaches simultaneously all primary and secondary lymphoid tissues where relatively high numbers of pDC are localized. This situation could explain why such viruses are able to induce a potent IFN-α response despite the activities of N^pro^ and E^rns^ [9].

Interestingly, it has been demonstrated that the virus not only needs a functional N^pro^, but also an E^rns^ with active RNase to establish persistent infections in cattle [14]. N^pro^ induces the degradation of IRF3 and thereby efficiently prevents IFN type I induction in all host cells including conventional DC, which have been induced to express IRF7 by IFN type I pre-treatment [19], [28]. However, N^pro^ can only partially prevent IFN-α responses in pDC [8] and is unable to stop the much more potent activation of pDC by infected cells (this study). We thus propose that E^rns^ has evolved to prevent this pathway of innate immune system activation, which is much more potent and therefore likely to be essential for the virus to be able to establish persistent infections, representing a main survival strategy of pestiviruses [46].

## Materials and Methods

### Ethics statement

Bleeding and care of donor pigs was carried out in accordance with EU standards and National laws (Tierschutzgesetz SR455). Specifically, approval of the protocol employed was obtained by the Animal Welfare Committee of the Canton of Bern, Switzerland (animal license BE26/11).

### Cell preparation and cell culture

The porcine kidney cell lines SK-6 [47], PK-15 (LGC Standards-ATCC, Molsheim, France) and the porcine immortalized endothelial cells PEDSV.15 [48](obtained from Dr. Jörg Seebach, University of Geneva, Switzerland) were propagated in Earle's minimal essential medium (MEM) substituted with 7% horse serum and in Dulbecco's minimal essential medium (DMEM) supplemented with 5% horse serum, nonessential amino acids and 1 mM Na-pyruvate, respectively. SK-6 cells stably expressing E^rns^ of CSFV strains Alfort/187, termed SK-6(E^rns^) were generated as described previously [49]. Baby Hamster Kidney (BHK) 21 cells were grown in Glasgow's minimum essential medium (Life Technologies) supplemented with 5% v/v fetal bovine serum (FBS, Biowest, Nuaillé, France). Peripheral blood mononuclear cells (PBMC) were obtained from blood of specific-pathogen-free pigs using ficoll-paque density centrifugation (1.077 g/L, Amersham Pharmacia Biotech). pDC were enriched as described previously [31] by cell sorting of CD172a^+^ PBMCs using the magnetic cell sorting system (MACS) with LD columns (Miltenyi Biotec GmbH, Germany). This permits a 10-fold enrichment of functional pDC to 2–5%. In some experiments, pDC were enriched by a first depletion of CD14^+^ monocytes followed by CD172a enrichment [24], permitting a pDC enrichment of around 5–10%. Enriched pDC were cultured in DMEM with Glutamax, 20 µm β-mercaptoethanol (Life Technologies). 10% FBS was only added to when indicated. Porcine monocytes were isolated by CD14^+^ selection using MACS with LS columns. Porcine MΦ were generated from CD14^+^ PBMCs as previously described using a 3-day culture in DMEM supplemented with 10% autologous porcine serum [50], [51].

### Viruses and replicons

CSFV strain vA187-1 was derived from the full-length cDNA clone pA187-1 [52]. Plasmid pA187-1 carries a full-length cDNA copy of the CSFV strain Alfort/187 and served as basis for all viruses and replicon cDNA constructs. The vA187-E^rns^Δ346 virus (referred to as CSFV-E^rns^Δ346 mutant), with a histidine deletion in E^rns^ at position 346 of the viral polyprotein resulting in loss of RNase activity [13] was rescued by standard procedure [52], [53] from plasmid pA187-E^rns^Δ346. This latter construct was generated from pA187-1 using PCR-based site-directed mutagenesis with oligonucleotide primers encompassing the deletion and PfuUltra DNA polymerase (Agilent), employing standard cloning techniques as previously described [54]. The plasmids pA187-ΔE^rns^ carrying an in frame deletion of the E^rns^ gene in the pA187-1 backbone, and pA187-D_136_N-ΔE^rns^ carrying the same deletion and expressing a non-functional D_136_N mutant of N^pro^ were described elsewhere [22], [49]. Plasmid pA187-E2del373 carrying an in frame deletion of the complete E2 gene in the pA187-1 backbone was also described previously [22], [49], [53]. Plasmid pA187-ΔApa encoding a CSFV replicon with a deletion of the structural protein genes C, E1, E^rns^ and most of the E2 gene (*i.e.* the codons encoding amino acids 96 to 962 of the polyprotein) was described earlier [53]. *In vitro* transcribed replicon RNA was produced using *SrfI*-linearized pA187-ΔE^rns^, pA187-D_136_N-ΔE^rns^, pA187-E2del373 or pA187-ΔApa as described [22], [49], [53]. VRPΔE^rns^ carrying a genome with a complete deletion of the E^rns^ gene were described previously, and produced by transfection of SK-6(E^rns^) cells with A187-ΔE^rns^ or A187-D_136_N-ΔE^rns^ replicon RNA. The SK-6(E^rns^) cells express the E^rns^ protein required for the generation of VRPΔE^rns^ by trans-complementation [22], [49]. Similarly, VRPΔE2 carrying the pA187-E2del373-derived replicon with a complete E2 deletion were rescued by transfection of SK-6(E2p7) cell line expressing the CSFV E2 and p7 proteins as described previously [22], [49], [53]. TGEV (TGEV; strain Perdue 115) was propagated in PK15 cells [55]. The FMDV type O UK/2001 isolate was grown in (BHK21) cells as described previously [56]. All virus titers were determined on SK-6 cells, PK-15 cells or BHK21 cells (for CSFV, TGEV and FMDV, respectively) by standard endpoint dilution and were expressed as 50% tissue culture infectious doses (TCID_50_) per ml.

### Quantitative real-time PCR

CSFV or VRP RNA was quantified using a published real-time RT-PCR [57]. Briefly, RNA was extracted using the Trizol method and RT-PCR performed with the SuperScript III Platinum One-Step qRT-PCR System (Life Technologies) using 7500 Real-time PCR System, Applied Biosystems. To determine the absolute number of RNA copies, vA187-1 RNA transcripts generated *in vitro* were employed.

### Generation of cell lines expressing E^rns^ using lentiviruses

A lentivirus (LV) expression system using plasmids obtained from the laboratory of Dr. Didier Trono (http://tronolab.epfl.ch/ Ecole Polytechnique Federale de Lausanne, Switzerland) or through Addgene (Cambridge MA, USA) [58], [59] was employed. For cloning of E^rns^ and its RNase-knock-out mutant variant into the lentiviral transfer plasmid pWPT-GFP (Addgene) the pA187-1 and pA187-E^rns^Δ346 plasmids were used as template for PCR amplification using primers to insert the MIuI and Sall restriction sites excising the GFP in the pWPT-GFP vector. The PCR products were first inserted into the pJET vector (Fermentas). The cloned E^rns^ genes of pJET-E^rns^ and pJET-E^rns^Δ346 were verified by nucleotide sequencing and then excised with MIuI and Sall and ligated into the pWPT-GFP vector employing standard techniques and Stbl2 bacteria, resulting in pWPT-E^rns^ and pWPT-E^rns^Δ346 respectively. All primer sequences and construction details can be obtained on request. In order to generate lentiviruses, HEK293T cells were transfected with the envelope plasmid (pMD2.G), the packaging plasmid (pCMV-R8.74) and the pWPT-E^rns^ or pWPT-E^rns^Δ346 plasmid using standard calcium phosphate precipitation. Medium was changed after overnight incubation at 37°C and the supernatant harvested after 48 h, centrifuged (350× g, 10 min) and filtered. The virus was purified and enriched by centrifugation on a 20% sucrose cushion at 100'000× g for 90 min at 4°C. The cells were transduced twice with 1∶100 dilutions of the purified lentiviruses in 1 ml serum free medium of a T25 cell culture flask followed by culture overnight at 37°C and medium change between the transductions. Transduction efficiency was found to be over 90% in terms of detectable anti-E^rns^ expression by flow cytometry and found to remain stable over at least three passages.

### Antibodies and flow cytometry

For pDC phenotyping and isolation, monoclonal antibodies (mAb) against CD172a (mAb 74-22-15A), CD14 (CAM36A) and CD4 (mAb 74-12-4 and PT90A) were used. Hybridomas for mAb 74-22-15A and 74-12-4 were kindly donated by Armin Saalmüller (University of Veterinary Medicine, Vienna, Austria). The mAb PT90A and CAM36A were purchased from VMRD (Pullman, WA). The hybridomas for mAb HC/TC26 [60] and C16 [61] binding the CSFV glycoprotein E2 and nonstructural protein NS3 respectively were kindly provided by Irene Greiser-Wilke, Hannover Veterinary School, Hannover, Germany. E^rns^ expression was demonstrated with mAb 140.1 (used at a 1∶200 dilution, Prionics, Switzerland) as described previously [62]. For NS3 and E^rns^ detection the cells were fixed and permeabilized with FIX&PERM solution (An der Grub Bio Research GmbH). As fluochromes, isotype-specific fluorescein isothiocyanate (FITC), R-phycoerythrin (RPE) (Southern Biotechnology Associates), RPE-Cy5 (Dako) and APC (Becton Dickinson, Basel , Switzerland) conjugates were used as described previously [63].

### Stimulation of pDC by infected cells

For stimulation of enriched pDC by cells infected with CSFV and VRP, SK-6 cells were infected at an multiplicity of infection (MOI) of 5 TCID_50_/cell, cultured for 24 h and then washed four times to remove the inoculums. Infectivity was verified and found to be above 90% NS3^+^ cells following infection with CSFV or VRP at these conditions. The SK-6 cells were then added at 40'000 cells/well for 96 well plate (if not indicated otherwise) or at 200'000 cells/well for 24-well plates. Freshly isolated CD172a^+^ enriched pDC were then added to the cultures at 200'000 cells/well of 96-well plates or 1×10^6^ per well of 24-well plates. After another 22 h, supernatants were isolated for IFN-α ELISA and the cells for NS3 expression in some experiments. For stimulation of pDC by cells infected with FMDV or TGEV virus the cells were infected at the indicated MOIs, incubated for 90 min at 37°C and washed 4 times before addition of enriched pDC. For FMDV BHK21 cells were employed, for TGEV PK15 cells. In some experiments we employed 24-well plate transwell inserts with 0.4 µm or 1.0 µm pore sizes (Corning, Sigma Chemicals, Buchs Switzerland and Becton Dickinson, Basel Switzerland). All cultures with enriched pDC were done at 39°C, 6% CO_2_. As a positive control for pDC stimulation, direct CpG D32 at 10 µg/ml [63] was used.

### RNase assay

A 50-mer RNA oligonucleotide probe complementary to nucleotides 12242-12193 of the vA187-1 genome sequence (GenBank accession number X87939.1) and carrying a Dyomics 781 modification at the 5′ end (Dy-781-O1-RNA) was synthesized by Dr. Fabian Axthelm (Microsynth AG, Balgach, Switzerland). The Dy-781-O1-RNA probe was mixed at 40 nM final concentration with MEM containing 3×10^−3^ U RNase A/ml as digestion control, with 50 mM TrisHCl pH 7.4 as negative control, and with the samples to be tested for RNase activity, and incubated for 1 h at 37°C. The treated probes were mixed with 2 volumes of 97% Formamide (Sigma) and separated on a 10% polyacrylamide and 35% urea gel in 133 mM TrisHCl, 45.5 mM boric acid and 3.2 mM EDTA. Image acquisition was performed with the Odyssey Infrared Imaging System (LI-COR).

### Inhibitors

As TLR7 inhibitor IRS661 (5′-TGCTTGCAAGCTTGCAAGCA-3′) and a control oligonucleotide (Ctrl-ODN; 5′-TCCTGCAGGTTAAGT-3′) was used [23]. IRS661 and Ctrl-ODN were purchased from Eurofins MWG Operon (Ebersberg, Germany). The impact of various metabolic inhibitors on SK-6 cells was tested by addition of the inhibitor for the last 2 h of infection, in order to avoid interference with infection. Before addition of pDC, the inhibitors were removed and the cells washed three times. The following final concentrations were used: 1 µM latranculin B, 5 µM nocodazole or 20 mM methyl-β-cyclodextrin (MβCD). All chemicals were purchased from Sigma Chemicals.

### IFN-α detection by ELISA and intracellular staining

IFN-α was quantified by enzyme-linked immunosorbent assay (ELISA) using the mAbs K9 and F17 (kindly provided by Dr. B. Charley, INRA, Jouy-en-Josas, France) as described previously [31]. For detection of IFN-α by intracellular staining mAb F17 with a previously published protocol was employed [63].

### Statistics

P values were calculated by an unpaired t-test using the GraphPad Prism Software.

## Supporting Information

Figure S1 **pDC but not monocytes represent the source of IFN-α after stimulation by infected cells.** pDC were purified by a combined depletion of CD14^+^ cells followed by CD172a enrichment. Monocytes were purified by CD14 cell sorting. Both pDC (10% purity) and monocytes (98% purity) were stimulated by MOCK or VRPΔE^rns^-infected SK-6 cells for 20 h. Intracellular IFN-α staining was then performed by three-color flow cytometry and IFN-α in the supernatants was quantified by ELISA. The results are representative of two independent experiments.
(TIF)

Figure S2 **Neutralizing antibodies prevent viral NS3 expression in pDC after stimulation by virions but not by infected cells.** Left panels: Enriched pDC infected with VRPΔE^rns^ (MOI of 5 TCID50/cell) in absence (upper left panel) or presence (lower left panel) of neutralizing serum. Right panels: the pDC were co-cultured with MOCK-treated or VRPΔE^rns^-infected SK-6 cells for 24 h, again in absence (upper panel) or presence neutralizing antibody. After 24 h, the cells were analyzed by three-color FCM to determine the NS3 expression in pDC (defined as CD4^+^CD172a^low^). Blue histograms represent mock cultures, red histograms cultures with VRP. The percentage of NS3^+^ pDC is shown.
(TIF)

Figure S3 **Viral protein expression in pDC after co-culture with VRPΔE^rns^-infected SK-6 or SK-6(E^rns^) cells.** Enriched pDC were co-cultured with MOCK-treated SK-6 cells, with CSFV- or VRPΔE^rns^-infected SK-6 cells, or with CSFV- or VRPΔE^rns^-infected SK-6(E^rns^) cells for 20 h as indicated, and then analyzed by three-color FCM to determine the NS3 expression in pDC (defined as CD4^+^CD172a^low^). The percentage of NS3^+^ pDC is shown. Mean and standard deviation calculated from triplicate cultures are shown. The results are representative of three independent experiments.
(TIF)

Figure S4 **E^rns^ does not inhibit virus replication.** Normal SK-6 cells, SK-6LV(E^rns^) or SK-6LV(E^rns^Δ346) cells were infected in A with VRPΔE^rns^ (MOI 5 TCID_50_/cell), in B with TGEV (MOI 0.01 TCID_50_/cell) or in C with FMDV (MOI 0.1 TCID_50_/cell). After 1 h, the inoculums were removed and the cells washed three times. In A, replication was determined by quantitative RT-PCR, in B and C by titration of virus in supernatants.
(TIF)

Figure S5 **SK-6(E^rns^) cells do not have an inhibitory effect on activation of pDC by CpG.** Enriched pDC were stimulated with CpG alone or co-cultured with different numbers of SK-6 or SK-6(E^rns^) cells. After 20 h, the IFN-α levels in the supernatants were quantified by ELISA. Mean and standard deviation calculated from triplicate cultures are shown.
(TIF)

Figure S6 **Viral replication is not affected after treatment of SK-6 cells with drugs.** SK-6 cells were infected with VRPΔE^rns^ for 24 h, washed and then treated with a DMSO control, nocodazole (5 µM), MβCD (20 mM) or latrunculin (1 µM) for 2 h at 37°C. The inhibitors were then removed and the cells washed three times and culture for a second period. At 6 and 24 h after drug treatment the cells were harvested. A. Viral RNA quantified by real-time RT-PCR. B. Viral NS3 expression determined by flow cytometry. The mean values of three replicates with standard deviation are shown.
(JPG)
