# Correction: Efficient Sensing of Infected Cells in Absence of Virus Particles by Plasmacytoid Dendritic Cells Is Blocked by the Viral Ribonuclease E^rns^

**DOI:** 10.1371/annotation/4290dfee-64fd-4157-89e3-8edbba912420

**Published:** 2013-08-06

**Authors:** Sylvie Python, Markus Gerber, Rolf Suter, Nicolas Ruggli, Artur Summerfield

1. The word "Blasmacytoid" is misspelled in the article title. The correct title is: Efficient Sensing of Infected Cells in Absence of Virus Particles by Plasmacytoid Dendritic Cells Is Blocked by the Viral Ribonuclease Erns. The correct citation is: Python S, Gerber M, Suter R, Ruggli N, Summerfield A (2013) Efficient Sensing of Infected Cells in Absence of Virus Particles by Plasmacytoid Dendritic Cells Is Blocked by the Viral Ribonuclease Erns. PLoS Pathog 9(6): e1003412. doi:10.1371/journal.ppat.1003412

2. In the funding statement, the word "Immunoprophylaxis" should instead read "Immunology".

3. In the legend title of Figure 1, "INF" should instead read "IFN".

4. In the second sentence of the legend body of Figure 4, the IRS661 was employed at 0.7 uM, instead of 1.7 uM. The legend body should instead read, "IRS661 inhibits pDC activation by direct CSFV or VRPΔErns infection (A) and by VRP-infected cells (B). In A, IRS661 was employed at 0.7 µM, and in B, at the indicated µM concentrations. In B, the IFN-α levels of the cultures treated with IRS661 were significantly lower (p<0.05) than those receiving control (CTRL) ODN at all concentrations. The bars represent the mean values of three replicates, with error bars showing the standard deviations. The results are representative of three independent experiments."

The below seven errors do not significantly affect the scientific understanding of the article.

5. The Results subheading "Drugs affecting cytoskeleton and membrane cholesterol prevent stimulation of pDC by infected cells." was incorrectly formatted, and should instead be formatted identically to the other Results subheadings.

6. In the legend body of Figure 1, the last sentence should include the word "was" so that it instead reads, "The purity of pDC was 2-5%."

7. In the second sentence of the Figure 6 legend body, "SK6" should instead read "SK-6".

8. In the penultimate sentence of the legend body of Figure S4, "inoculums" should instead read "inocula".

9. In the last sentence of the first paragraph of the Results, "SK6" should instead read "SK-6".

10. In the second paragraph of the Discussion section, in the sentence reading "Only at this acidic location the RNAse function...", "RNAse" should instead read "RNase".

11. In the first sentence of the seventh paragraph of the Materials and Methods section, under the subheading "Stimulation of pDC by infected cells", "inoculums" should instead read "inocula".

