# Supplementary figures and images for: Efficient Sensing of Infected Cells in Absence of Virus Particles by Blasmacytoid Dendritic Cells Is Blocked by the Viral Ribonuclease Erns
Source: PLoS Pathog. 2013 Jun 13;9(6):e1003412. doi: 10.1371/journal.ppat.1003412 (PMC3681750; doi:10.1371/journal.ppat.1003412)

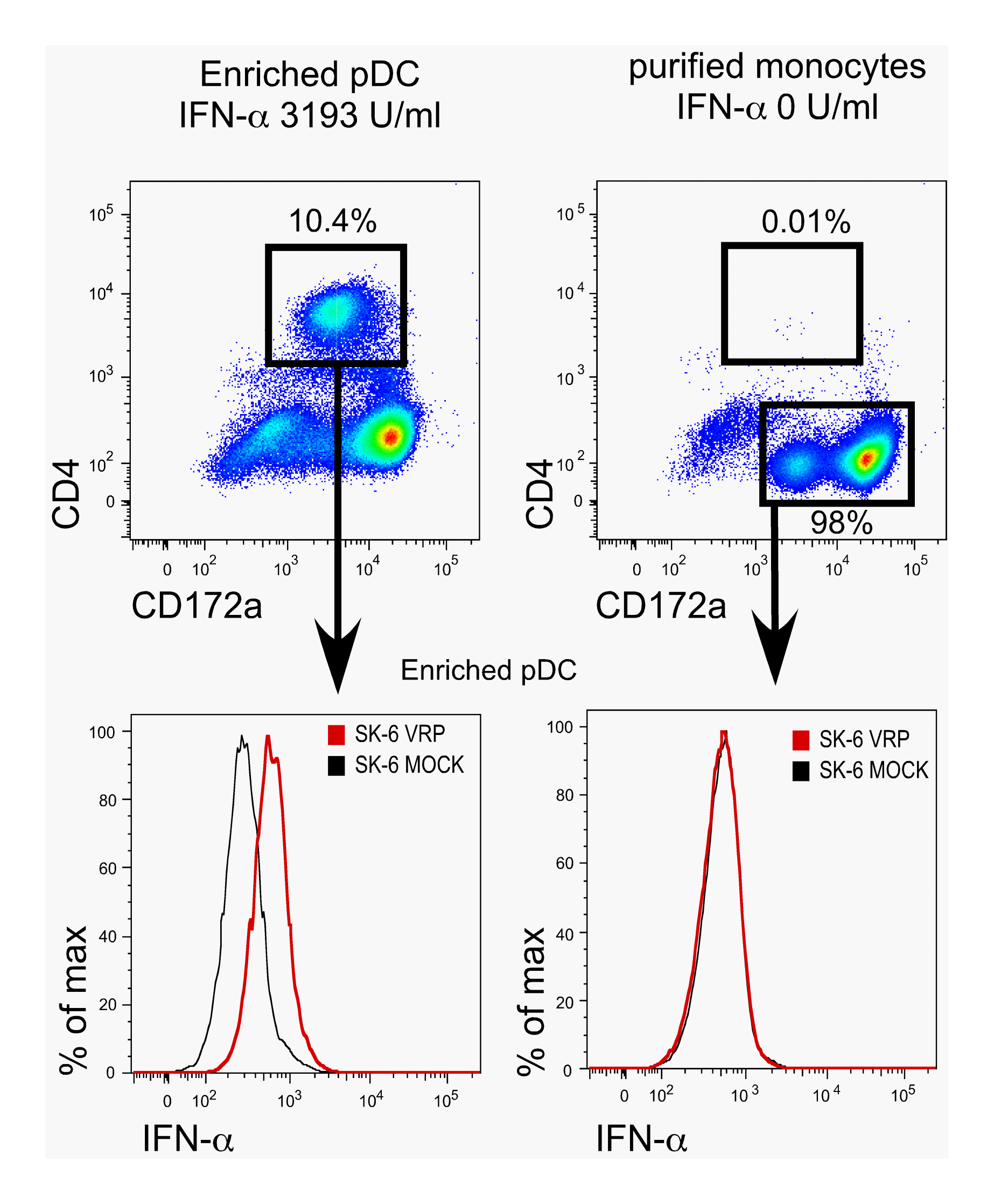

Supplement: Figure S1 — pDC but not monocytes represent the source of IFN-α after stimulation by infected cells. pDC were purified by a combined depletion of CD14+ cells followed by CD172a enrichment. Monocytes were purified by CD14 cell sorting. Both pDC (10% purity) and monocytes (98% purity) were stimulated by MOCK or VRPΔErns-infected SK-6 cells for 20 h. Intracellular IFN-α staining was then performed by three-color flow cytometry and IFN-α in the supernatants was quantified by ELISA. The results are representative of two independent experiments. (TIF) [file ppat.1003412.s001.tif]

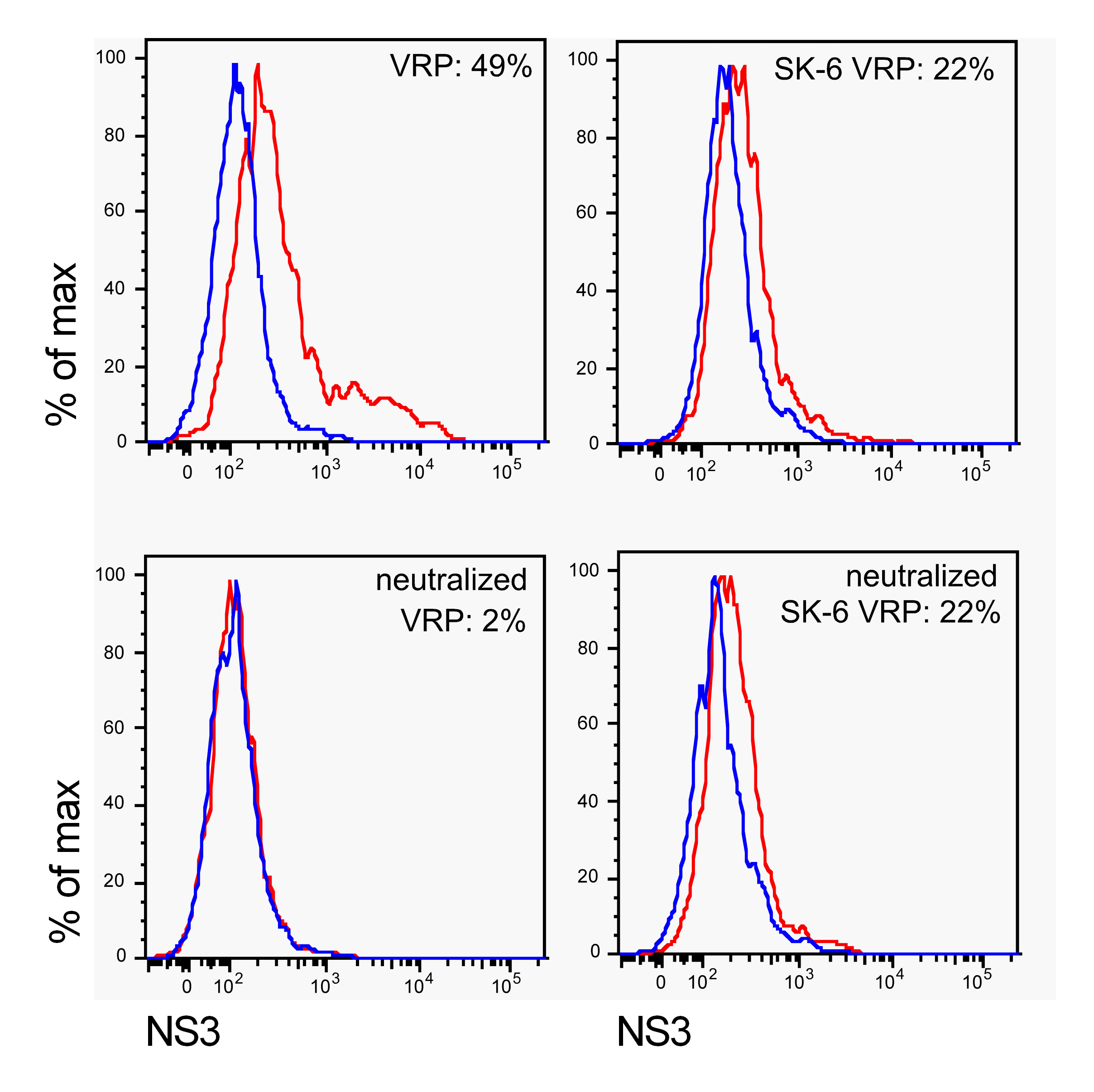

Supplement: Figure S2 — Neutralizing antibodies prevent viral NS3 expression in pDC after stimulation by virions but not by infected cells. Left panels: Enriched pDC infected with VRPΔErns (MOI of 5 TCID50/cell) in absence (upper left panel) or presence (lower left panel) of neutralizing serum. Right panels: the pDC were co-cultured with MOCK-treated or VRPΔErns-infected SK-6 cells for 24 h, again in absence (upper panel) or presence neutralizing antibody. After 24 h, the cells were analyzed by three-color FCM to determine the NS3 expression in pDC (defined as CD4+CD172alow). Blue histograms represent mock cultures, red histograms cultures with VRP. The percentage of NS3+ pDC is shown. (TIF) [file ppat.1003412.s002.tif]

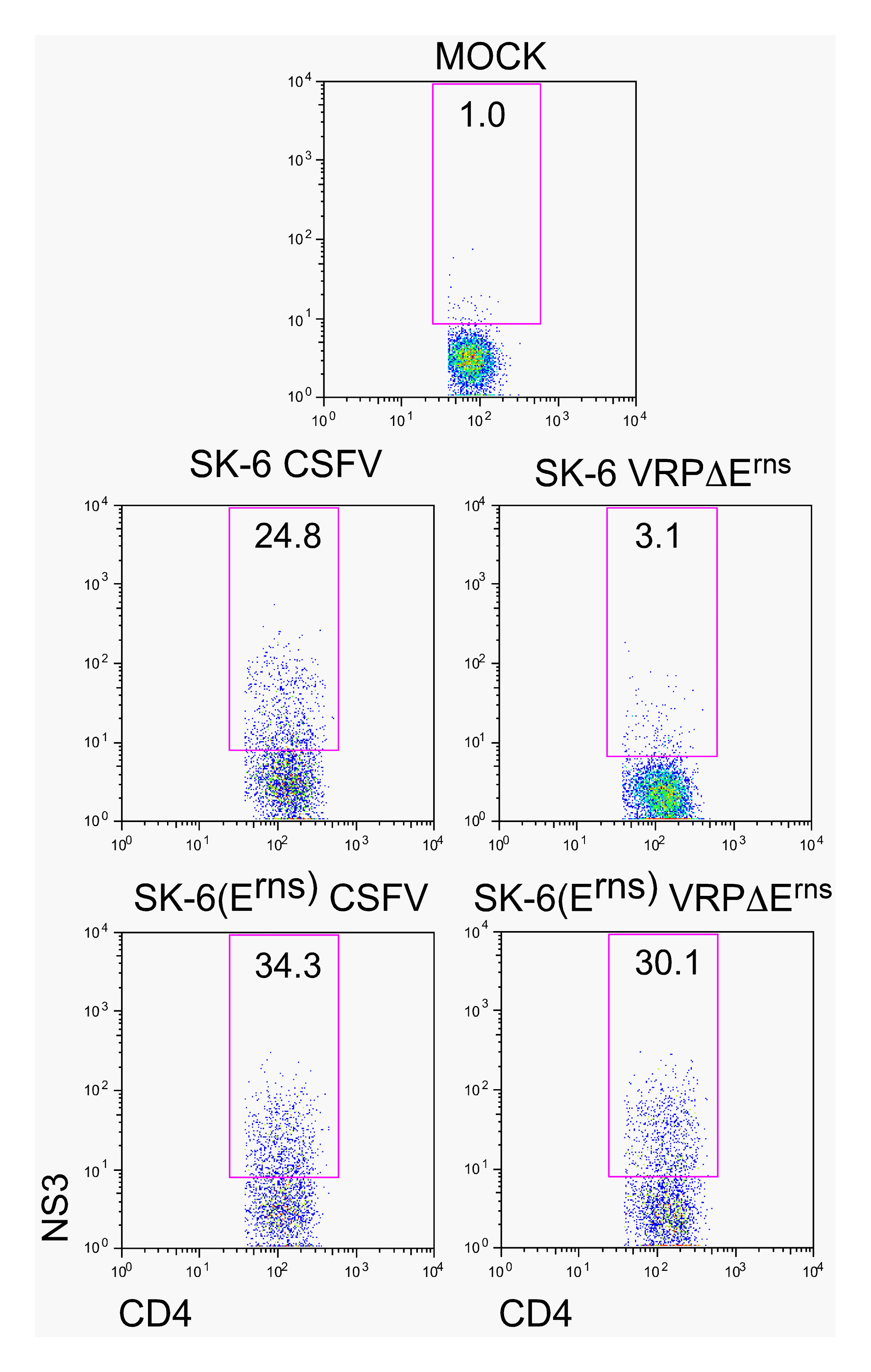

Supplement: Figure S3 — Viral protein expression in pDC after co-culture with VRPΔErns-infected SK-6 or SK-6(Erns) cells. Enriched pDC were co-cultured with MOCK-treated SK-6 cells, with CSFV- or VRPΔErns-infected SK-6 cells, or with CSFV- or VRPΔErns-infected SK-6(Erns) cells for 20 h as indicated, and then analyzed by three-color FCM to determine the NS3 expression in pDC (defined as CD4+CD172alow). The percentage of NS3+ pDC is shown. Mean and standard deviation calculated from triplicate cultures are shown. The results are representative of three independent experiments. (TIF) [file ppat.1003412.s003.tif]

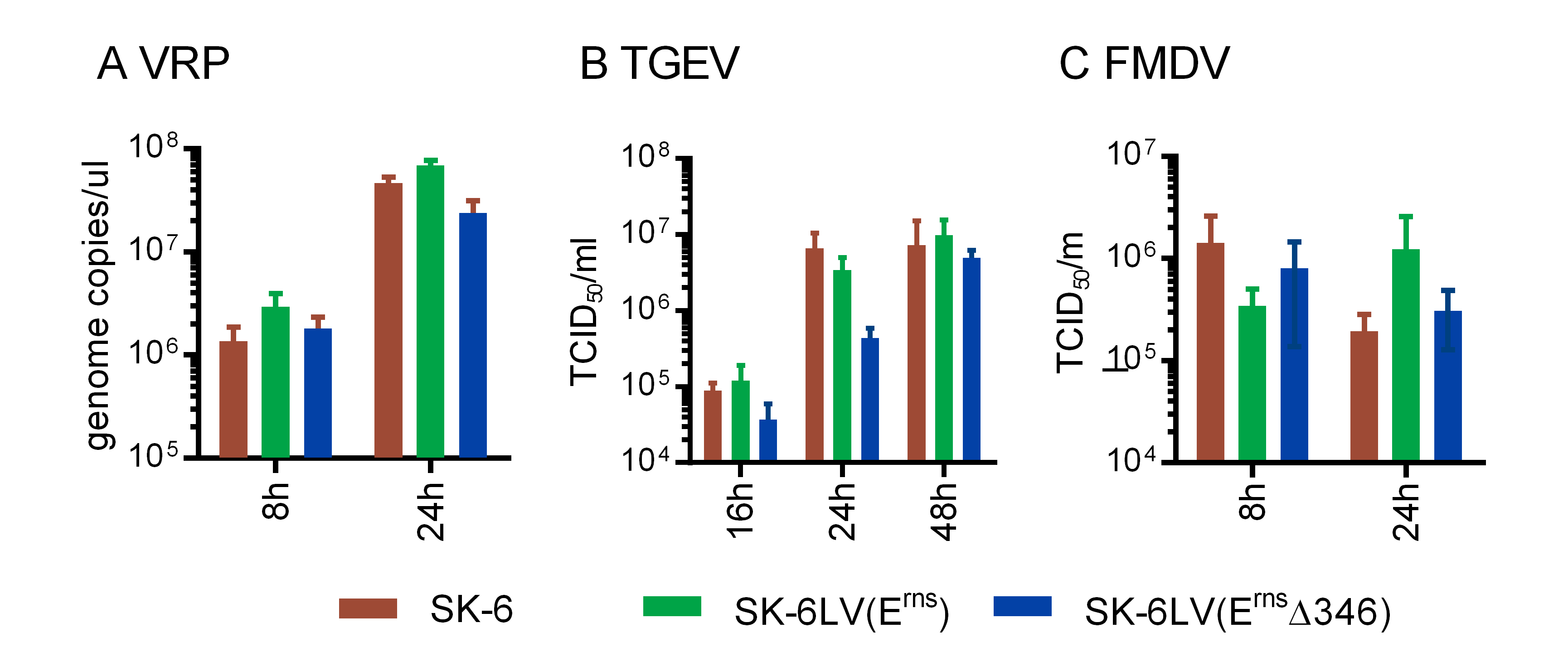

Supplement: Figure S4 — Erns does not inhibit virus replication. Normal SK-6 cells, SK-6LV(Erns) or SK-6LV(ErnsΔ346) cells were infected in A with VRPΔErns (MOI 5 TCID50/cell), in B with TGEV (MOI 0.01 TCID50/cell) or in C with FMDV (MOI 0.1 TCID50/cell). After 1 h, the inoculums were removed and the cells washed three times. In A, replication was determined by quantitative RT-PCR, in B and C by titration of virus in supernatants. (TIF) [file ppat.1003412.s004.tif]

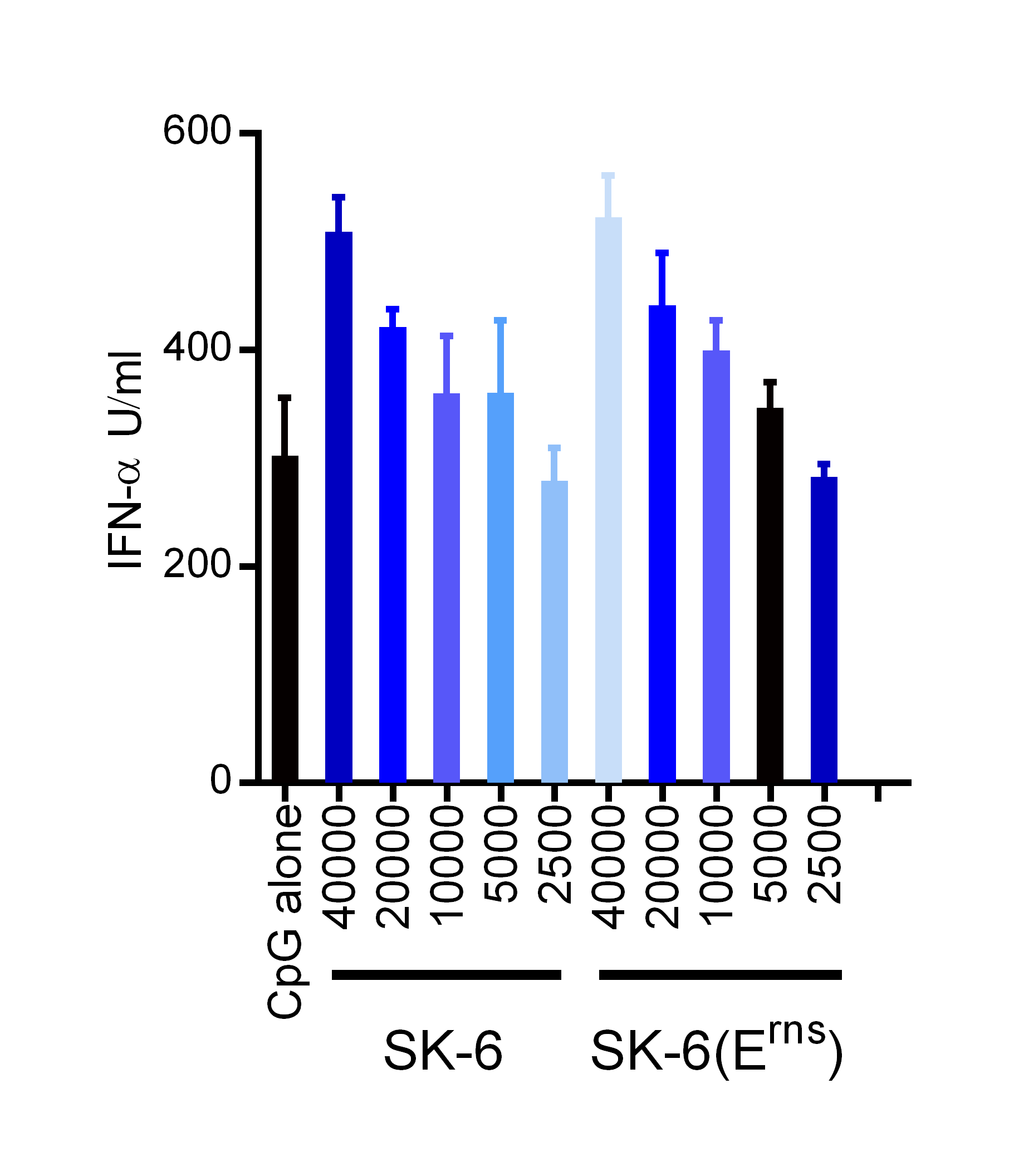

Supplement: Figure S5 — SK-6(Erns) cells do not have an inhibitory effect on activation of pDC by CpG. Enriched pDC were stimulated with CpG alone or co-cultured with different numbers of SK-6 or SK-6(Erns) cells. After 20 h, the IFN-α levels in the supernatants were quantified by ELISA. Mean and standard deviation calculated from triplicate cultures are shown. (TIF) [file ppat.1003412.s005.tif]

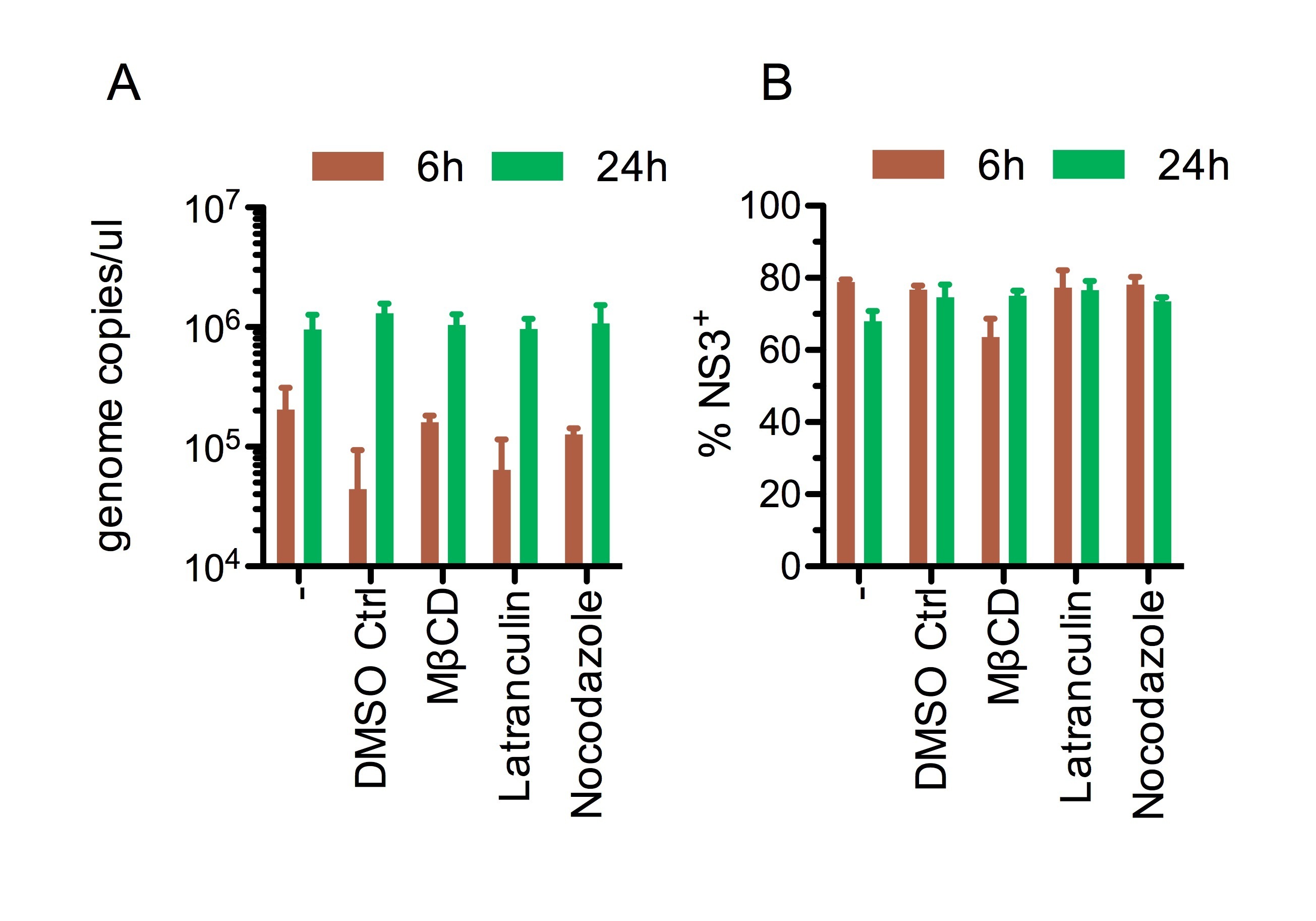

Supplement: Figure S6 — Viral replication is not affected after treatment of SK-6 cells with drugs. SK-6 cells were infected with VRPΔErns for 24 h, washed and then treated with a DMSO control, nocodazole (5 µM), MβCD (20 mM) or latrunculin (1 µM) for 2 h at 37°C. The inhibitors were then removed and the cells washed three times and culture for a second period. At 6 and 24 h after drug treatment the cells were harvested. A. Viral RNA quantified by real-time RT-PCR. B. Viral NS3 expression determined by flow cytometry. The mean values of three replicates with standard deviation are shown. (JPG) [file ppat.1003412.s006.jpg]
